# Pathways to Wellbeing: Reconceptualizing Resilience to Foreground Marginalized Teachers’ Agentic Resistance

**DOI:** 10.3390/bs15121603

**Published:** 2025-11-21

**Authors:** Ji Hong, Lijie Liu, Yijia Chen, Soojeong Lee, Jing Zhao, Travis Dean, Taylor Roloff

**Affiliations:** Department of Educational Psychology, College of Education, University of Arizona, 1430 E. 2nd Street, Tucson, AZ 85721, USA; lijieliu@arizona.edu (L.L.); yijiachen@arizona.edu (Y.C.); sjlee23@arizona.edu (S.L.); jingzhao1@arizona.edu (J.Z.); travisdean@arizona.edu (T.D.); taylorroloff27@arizona.edu (T.R.)

**Keywords:** marginalized teachers, agentic resistance, teacher resilience, teaching wellbeing, critical qualitative inquiry

## Abstract

This study reconceptualizes resilience by centering marginalized teachers’ agentic resistance as a critical pathway to wellbeing. Using critical qualitative inquiry, we conducted in-depth interviews with 17 U.S. teachers who identified with marginalized racial, gender, and/or sexual identities to explore how they resist structural oppression and sustain their professional and personal identities. Findings revealed that resistance emerged in various ways, including inclusive curriculum design, confrontation with colleagues or administrators, embodied identity expression, and support drawn from contexts and resources for resistance. These resources included social movements and ancestors’ legacies, demonstrating that resistance is not merely individual coping but a collective, identity-affirming practice. We argue that resilience must be reframed to include intentional and proactive resistance, which enables marginalized teachers to challenge oppressive school structures, promote educational equity, and sustain their own flourishing. This study offers a critical framework that shifts resilience from passive endurance to politically engaged practice, emphasizing the need for institutional supports that empower marginalized teachers to resist, persist, and thrive in inequitable educational systems.

## 1. Introduction

In the current American education system, the racial structural mismatch between the teachers and the students has become an important issue that requires attention. Although the student group is increasingly diverse, the growth in teacher diversity is extremely slow and even regresses in some ethnic groups (Goldhaber et al., 2019). For example, in K–12 public schools in the United States, students of color are expected to make up 56% of enrollment by 2030 (National Center for Education Statistics, 2022; TNTP, 2024). However, only 22.4% of teachers are people of color, resulting in a student-to-teacher-of-color ratio as high as 36.5:1 (compared to 8.5:1 for White students to White teachers), and in 97% of districts, teacher diversity remains lower than student diversity (TNTP, 2024). This imbalance leads to a large number of students of color rarely being exposed to teachers of similar race throughout their education, thereby weakening students’ sense of belonging, learning motivation, and long-term development potential (Redding, 2019).

The racial diversity of the teacher not only helps improve students’ academic performance and mental health, but it also breaks the stereotypes that White students have towards people of color and promotes cross-racial understanding and mutual respect (Goings et al., 2018; Milner, 2011). Students are also more likely to receive recognition and support from teachers who share their same racial background (Lindsay & Hart, 2017) and less likely to be subject to disciplinary actions due to cultural misunderstandings (Gregory & Mosely, 2004; McKown & Weinstein, 2002). Furthermore, racial matching may promote communication between family and school, enhance parental participation, and further expand students’ access to educational resources (Redding, 2019). Therefore, teacher diversity not only supports students’ development academically and personally but also serves as an important strategy to promote educational equity.

Similarly, research consistently shows that lesbian, gay, bisexual, transgender, and queer (LGBTQ+) teachers positively influence students’ sense of belonging, willingness to seek support, and perceptions of a supportive classroom climate, which are closely connected to mental health and academic engagement (Busch et al., 2024; GLSEN, 2021). For LGBTQ+ students in particular, these educators provide crucial support by demonstrating identity and experience, which helps alleviate loneliness and stress during identity recognition, enhances self-esteem, and promotes positive psychological development (GLSEN, 2021; Leonard, 2022; Posner, 2024). The diversity of such teachers brings new perspectives and experiences to all students. It can enhance understanding and empathy among peers, help heterosexual and cisgender students broaden understandings of gender and diversity, challenge stereotypes, and thereby promote deeper understanding and respect (Leonard, 2022; Linley et al., 2016). Therefore, diversity supports students’ holistic development and advances educational equity and multicultural education.

However, despite the importance of having diverse teachers in schools, marginalized teachers’ turnover rates are significantly higher than that of privileged teachers (e.g., White, cisgender, heterosexual) (Ingersoll & May, 2011; Ingersoll et al., 2021). This is inseparable from the fact that teachers with marginalized identities, specifically those who identify as Teachers of Color and LGBTQ+ teachers, often work under conditions of systemic inequities, oppression, and isolation (Castro-Gill, 2025; J. Hong et al., 2024a; Wright-Mair, 2023). These challenges contribute to emotional strain, professional instability, diminished wellbeing, and high turnover rates (J. Hong et al., 2024b; National Center for Education Statistics, 2022). In order to truly achieve educational equity, we need to address the pressures and challenges faced by these teachers rather than their resilient nature alone. In particular, it is important to examine how they rebuild and maintain their sense of wellbeing within the oppressive structure, enabling them to advocate for themselves and their students. Teachers with a strong sense of wellbeing often feel a sense of belonging and respect within the school. They can freely express their racial and cultural identities and grow across multiple dimensions, including psychological, emotional, relational, and professional aspects (Day & Gu, 2010; Deci & Ryan, 2008; Hascher & Waber, 2021). We recognize that one of the pathways to wellbeing is resilience, which has been commonly regarded as a capacity to help teachers navigate and manage these adversities (e.g., Robinson & Schmitz, 2021; Stieglitz, 2010). However, we also recognize limitations of the current resilience framework, which is often understood as an individual trait (Tait, 2008). This limited perspective risks overlooking systemic oppression and inequities. Thus, in this study, we attempt to reconceptualize resilience to expand resistance, which enables marginalized teachers to challenge and transform oppressive conditions. We aim to explore the ways marginalized teachers enact resistance to advance educational equity, as well as the contextual conditions that empower them to harness their agency for resistance.

## 2. Theoretical Framework

Resilience has been traditionally understood as a stable, internal, individual trait that enables people to “bounce back” from hardships (Masten et al., 1990; Patterson, 2002). This conceptualization has evolved over the years to address resilience not as an innate, fixed quality, but as a capacity that encompasses multiple dimensions, such as personal resources (e.g., efficacy, sense of vocation), contextual supports (e.g., relational dynamics), coping strategies (e.g., ways to solve problems), and outcomes (e.g., job satisfaction, belonging) (Beltman et al., 2011; Mansfield et al., 2016). In particular, teacher resilience research has emphasized two key perspectives, which include (1) resilience as a latent capacity, which can fluctuate in relation to various internal and external sources, and (2) the explanatory power of resilience on teachers’ wellbeing, retention, and effectiveness in teaching (e.g., Castro et al., 2010; Chen, 2024; Day & Gu, 2014; Day & Hong, 2016).

Rather than being viewed as a stable trait, resilience is now understood as a capacity that allows individuals to actively navigate adversity and demonstrate positive outcomes in the face of challenges. This capacity reflects the human potential to continually adjust to and overcome personal vulnerability and environmental stress, enabling individuals to bounce back and maintain physical and mental health when confronted with risks (Newman, 2005; Wuest & Subramaniam, 2021). This adaptive capacity is shaped by the dynamic interaction between individuals and their environments as they continuously adjust to changing personal and external stressors (Kowitarttawatee & Limphaibool, 2022; Shin et al., 2012). For instance, studies conducted in South Australia and Western Australia (Johnson & Down, 2013; Johnson et al., 2015) showed that teacher resilience is influenced by five key conditions: interpersonal relationships, school culture, teacher identity, teacher work, and policies and practices.

Scholars have also emphasized resilience as a dynamic and fluctuating capacity that fosters positive adaptive outcomes for teachers (Chen, 2024; Gu & Day, 2013; Gu & Li, 2013). As such, it is widely regarded as essential for teachers to sustain their wellbeing, teaching effectiveness, job satisfaction, and retention in their profession (e.g., Day & Gu, 2013; Mansfield et al., 2016). At the organizational level, supportive behaviors of school leaders and sufficient working resources can effectively enhance teachers’ multi-dimensional resilience in professional, emotional, and social aspects, thereby strengthening teachers’ educational leadership and professional commitment (Bagdžiūnienė et al., 2022). At the individual level, teacher resilience is not only positively correlated with psychological variables such as happiness, quality of life, self-efficacy, and emotional regulation, but also negatively correlated with depression, stress, anxiety, and burnout (Baguri et al., 2022; Brouskeli et al., 2018; Cho et al., 2021; Pečjak & Pirc, 2022). These findings highlight that resilience plays a key role in individual career adaptation and mental health. Teachers with a strong sense of resilience are better equipped to manage stress, sustain their motivation, and maintain strength in the face of adversity (e.g., Bobek, 2002; Castro et al., 2010). On the contrary, teachers with a weaker sense of resilience are at higher risk for burnout, emotional exhaustion, loss of motivation, and attrition (Chang, 2009; J. Y. Hong, 2012).

Although research on teacher resilience has provided valuable insights into teachers’ work and lives, notable limitations remain due to the absence of a critical perspective of the oppressive conditions that create the need for resiliency. Despite acknowledging contextual influences, resilience often focused on individual actors and their relational dynamics in schools (e.g., Day & Gu, 2013; Jordan, 2012). For example, Mackrain (2013) measured adult resilience from individuals’ relationships, beliefs, initiative, and self-control, still framing it primarily as an individual trait. Similarly, Glazzard and Rose (2019) regarded resilience as an ability to help individuals recover when facing challenging situations. Such perspectives tend to emphasize how teachers dealt with challenges and how they “bounced back”, attaching importance to identifying risk and protective factors for teachers’ resilience development, while considering coping as an individual responsibility (Howard & Johnson, 2004; Johnson & Down, 2013).

Later studies acknowledged that resilience is influenced and shaped by both personal characteristics and social ecology and contextual factors (Oldfield & Ainsworth, 2022; Ungar et al., 2013). Despite this recognition, these works still focused on developing coping strategies and tended to frame schools and communities as sites of protective factors and coping resources. In addition, they did not address the additional burdens and oppressions faced by marginalized teachers, therefore overlooking the risk factors that stem from the systemic inequities. This lack of attention to systemic inequities posed a danger of assuming that resilience only refers to assimilating into dominant culture and norms, rather than confronting systemic and structural inequities (Duggan, 2003; Halberstam, 2011). This is especially problematic for marginalized teachers whose social identities are not aligned with the norms and values of the school and society (e.g., Teachers of Color, LGBTQ+ teachers). To address the challenges caused by this misalignment, several studies investigated marginalized teachers’ teaching lives and their strategies for coping with identity-related challenges.

Drawing on Critical Race Theory, Hernández-Johnson et al. (2023) argued that teacher resilience cannot be separated from racialized school contexts and the structural forces that undermine wellbeing. Consequently, resilience should entail navigating oppressive environments while refusing to assimilate into white-normed expectations and heteronormativity, which leads to proactive resistance actions. Kirmaci (2023) further addressed the importance of resistance in resilience development by engaging with the concept of transformational resistance. Solorzano and Bernal (2001) introduced transformational resistance and expanded beyond self-defeating models of resistance by centering practices that directly confront systemic injustice. Using this framework, Kirmaci examined the lived experiences of a marginalized teacher, illustrating how they went from resilience to resistance. Additionally, Casado Pérez (2019) emphasized daily resistance strategies as practices that affirm identity, build community, and resist institutional marginalization by using Critical Race Feminist Theory. Love and Hancock (2022) addressed the importance of driving teachers to transform power dynamics and contribute to a more equitable environment for marginalized teachers based on Disability Critical Race Theory (DisCrit).

Existing studies show that resilience is closely linked to individuals’ wellbeing, with resilience strategies often contributing to positive outcomes (Liu et al., 2022; Paredes et al., 2021; Peel et al., 2023). However, these frameworks tend to overlook how systemic inequities, such as racism, cultural taxation, and institutional oppression, impact teachers’ daily lives and struggles. For marginalized teachers, this gap is critical as such inequities undermine not only their wellbeing but also their sense of self as teachers (Pizarro & Kohli, 2020; Smith et al., 2007). Therefore, we reconceptualize resilience as a concept of resistance. Resistance emphasizes teachers’ agency and proactive efforts to confront oppressive structures. By resisting assimilation and asserting professional and cultural identities, teachers preserve their sense of agency, strengthen connections that reduce isolation, and cultivate competence in their practice. In this way, resistance functions not only as a strategy for challenging injustice, but also as a foundation for teachers’ wellbeing and identity, enhancing their professional purposes and capacity to thrive (Hascher & Waber, 2021; Robinson & Schmitz, 2021).

Thus, reconceptualizing marginalized teachers’ persistence in navigating adversity (i.e., resilience) as resistance provides a more critical and transformative lens. Resistance is not only opposition but deeply agentic. It involves recognizing and naming injustice, asserting professional and cultural identities, and creating space for equity within systems that often erase or devalue marginalized voices. Instead of conforming to injustice or adapting to the dominant culture, marginalized teachers should be able to actively resist and disrupt masternarratives such as white supremacist ideologies, colonialism, and heteronormativity, to stay true to their sense of self and genuinely thrive (Robinson & Schmitz, 2021).

Recognizing the need to reconceptualize resilience to encompass marginalized teachers’ resistance in a way that fosters a more equitable educational environment, we proposed the following research questions:

1. How do marginalized teachers experience resistance in relation to educational inequity?
2. Where do marginalized teachers draw on their strengths to exercise agency to resist?

## 3. Materials and Methods

We employed critical qualitative inquiry, which focuses on exposing and challenging systems of oppression and inequity through individuals’ constructed accounts of their experiences and meanings (Cannella & Lincoln, 2015; Denzin, 2016). Thus, grounded in a critical paradigm, the main focus of this design is (i) to capture a detailed examination of marginalized teachers’ lived experiences, (ii) to understand how these teachers make sense of their experiences, and (iii) to unpack the school, district, community, and larger societal contexts and their power dynamics, which privilege certain groups while marginalizing others (Call-Cummings & Dazzo, 2023). Also, this study was conducted in accordance with the Declaration of Helsinki, and the protocol was approved by the Institutional Review Board of the University of Oklahoma (Project identification code #13036, Approval date: 11 February 2021).

The research team comprised seven members, each of whom possesses marginalized identities (e.g., Asian, Queer, Woman) and identifies as allies of LGBTQ+ communities, advocating a critical paradigm. Considering these backgrounds, we have encountered invisibility and silenced voices ourselves, which has heightened our appreciation for the significance of amplifying marginalized voices and fostering resilience, resistance, and wellbeing. We also believe that in order to support and enhance the wellbeing of marginalized teachers, it is crucial to challenge dominant narratives such as white supremacy and heteronormativity, which are profoundly embedded within social, cultural, and historical frameworks.

### 3.1. Participants and Data Sources

Marginalized teachers were recruited nationwide via social media postings and listservs of various teacher organizations in the U.S. A total of 17 teachers who identify as marginalized in terms of race, gender, and/or sexuality participated. As shown in Table 1, their background varied across all three dimensions (All names used in this paper are pseudonyms). Prior to the data collection, informed consent was obtained from each participant. Semi-structured, in-depth individual interviews were conducted via an online platform. Interview questions captured participants’ transactional experiences as they recognized adversities within the school and wider societal power structures, and took actions to resist and to advocate for their students and themselves. Specifically, the interview protocol encompassed five domains of questions (i) participants’ background (e.g., school context, personal social identities), (ii) interpersonal challenges with students, colleague teachers, school administrators, and students’ parents, (iii) instructional challenges (e.g., curriculum design, teaching plan development, instructional delivery), (iv) resources they draw in the face of challenges, and (v) their impact on participants’ wellbeing. Based on participants’ responses, we followed up with probing questions to further unpack details. Each interview lasted approximately 90 to 110 min and was audio-recorded through the online platform.

### 3.2. Data Analysis and Trustworthiness

Interviews were transcribed verbatim and analyzed inductively Via open-coding (Miles & Huberman, 1994). Codes reflected conditions, interpersonal dynamics, and implicit or explicit power relations and the roles they played in participants’ resistance to school- and community-based norms and values (Wolgemuth, 2014). Each researcher coded each transcript independently, and then once all interviews were coded by all researchers, we met as a group to discuss similarities and differences in our codes. Whenever there were differences, we discussed the sources of differences and reconciled them for triangulation, which helped us develop a shared understanding of the data (Creswell & Poth, 2016). Those reconciled codes across all participants’ interviews were then compared, contrasted, and sorted to generate categories. Those categories were further examined to search for linkages in the emergent data structure, to form dominant thematic patterns, and to develop interpretive accounts. Researchers continued to be engaged in collaborative discussions throughout the analytic processes.

Guided by critical qualitative inquiry, data saturation was determined based on the achievement of depth and complexity when additional interviews no longer generated new insight into how marginalized teachers resist structural oppression and sustain their identities (Fusch & Ness, 2015). We collected and analyzed data concurrently through iterative coding and reflexive memoing, which enabled emerging patterns to be compared against subsequent narratives. After 17 participants, new interviews confirmed and elaborated the established themes without generating novel categories, which means data saturation was achieved through analytic completeness. Also, in order to enhance trustworthiness of the study, we employed several key strategies such as member checking and peer debriefing (Patton, 2002). When themes were shared to the participants, not all responded or provided feedback; however, three participants concurred that the themes accurately represented their experiences.

## 4. Findings

Findings revealed that marginalized teachers actively exercised their agency to resist inequitable educational practices, which included multiple dimensions of activities such as curriculum choice, teaching practice, interpersonal confrontation, and bodily expression of their identities. Additionally, their resistance was supported by resources drawn from larger social movements, colleague support, and ancestral strength.

### 4.1. Multifaceted Act of Resistance

#### 4.1.1. Curriculum Choice and Teaching Practice to Foster Critical Consciousness

All marginalized teachers participated in this study consciously chose to resist through their curricula and intentional efforts to incorporate inclusive materials that support marginalized students. For instance, Alma, a 4th grade bisexual teacher who teaches all subjects, challenged dominant historical narratives by paralleling the stories of Susan B. Anthony and Clara Luper in her lessons. She highlighted the limitations of Anthony’s activism that excluded women of color.

*I included like a Susan B. Anthony, lesson and I also did a Claire Luper. And with Clara Luper of course, like it talks about the citizens, and it’s about race, and Susan B. Anthony, it’s just fighting for women to vote. But with Susan B, Anthony, that lesson I also included, like, some slides that talked about how she [Claire Luper] was inclusive and fighting for other people besides White women.*

Similarly, Edenia, a 4th grade Latina lesbian teacher who teaches all subjects, also shared her deliberate and conscientious effort to utilize language that advances gender equity within her classroom.

*I don’t use pink and blue. Pink for girls and blue for boys. I use purple for boys and teal for girls. So just in terms of like making sure that my classroom is the open space that I would have always wanted as a kid. Yeah, just the language is super super intentional because you never know what a student is struggling with for sure.*

Edenia demonstrated her intentional act of resisting master narratives by integrating social justice issues and systemic inequities into her science lessons. When teaching students about electrical systems, including their components and functions, she deliberately compared these electrical systems to wider societal systems.

*I talked to them about how systems aren’t just relevant in science. Systems are also relevant in society. So we have systemic racism that’s embedded into policies that turn into racist policies and perpetuate racist ideas. And that’s also systemic misogyny and systemic sexism for women. You think about like access to opportunities for people that are people of color. We also like, there’s also like systemic challenges for people who are LGBTQ, for people who are women, for people who are native, indigenous, Black, indigenous people of color.*

Teachers’ efforts to “make my curriculum decolonize” and to “infuse diversity and culture into any subject” were often linked to their endeavor to help students see the inequities and systemic oppression in the real world by fostering critical consciousness. Jelani, a 9th grade Black bisexual social science teacher, emphasized her role as an educator in cultivating students’ awareness of systemic injustice and nurturing them to act as socially responsible individuals. She explicitly noted the importance of “developing a critical awareness among the students and being able to understand the realities that [are] being taught in class, so that they can be in a position to practice whatever they have been taught in class and outside.” As such, marginalized teachers challenged prevailing societal norms and values by designing or utilizing inclusive and justice-oriented curricula and by nurturing students’ critical consciousness.

#### 4.1.2. Confronting Colleagues and Administrators

When marginalized teachers faced unfair treatment or prejudice from their colleagues or school leaders, they fearlessly confronted them. Although not all participants’ transcripts included this theme, about two-thirds of them mentioned this act of resistance. Shanika, a 6th through 8th grade Black English Language Arts (ELA) teacher, challenged the school librarian who refused to teach her class because of its racial composition. She confronted the teacher directly.

*The library teacher, she didn’t want to teach the kids in my class, because there were too many Black kids in my class. And so I’m like, listen, you’ve missed my class several times for real, you need to come to teach my students, and they better not have S’s on the report cards…She’s like, ‘Are you threatening me?’ I was like, ‘No, I’m promising you what needs to happen. And I will make sure that everyone knows what’s happening if I don’t see this corrected.’*

Edenia also commented on how she confronted colleague teachers who “don’t really feel creating an inclusive environment is important”.

*But just because I’m teaching that [equity for all students] in my classroom doesn’t mean other teachers are teaching in their classroom. So they say, oh, so-and-so from so-and-so is class called me gay. And so then I have to go and talk to those teachers and say, hey, like you need to have this conversation with that student… because we need to make this a safe and positive environment for everybody.*

Even if these confrontations created tensions with colleagues and may make them as a “troublemaker” to others, they prioritized advocating for students’ safety, wellbeing, and sense of belongingness in their school spaces.

However, when this confrontation occasionally extended to interactions with administrators, it was more difficult to address due to the prevailing power dynamics. When Amira, a 9th through 12th grade Afro-indigenous Latina math teacher, was observed by her administrator, the administrator was dissatisfied with her teaching approach. She used the story and characters in a movie that is well-connected to students’ background and culture, who are dominantly Black students, but her administrator wanted Amira to use White character names, instead of Black names. As a result, he assigned her low evaluation scores. Amira explained this incident:

*I’m responsive to what’s going on right now in the culture of this country. And he just like really gave me poor scores. And I just felt like, you know what, I am threatening his space. And he’s grading me based upon that threat versus what it is. And so I did write a rebuttal to his observation.*

Amira’s act of writing a rebuttal to an unfair teaching evaluation exemplifies her courage and agency to stand up for herself and express her concerns with confidence.

#### 4.1.3. Embodied Expression of Their Identities

Marginalized teachers’ resistance also included how they communicated their identities through physical bodies and actions. About one-third of the participants mentioned this theme. For instance, Makala, an 11th and 12th grade Black English teacher, resisted the norms of school and community regarding the bias that straightened hair “looks more professional” than her natural hair. Makala rejected the idea and resisted by communicating her Black identity without suppression.

*I don’t feel the need to straighten my hair for graduation. I don’t think that it’s unprofessional… Everyone else gets to wear their hair the way it naturally comes out of their head—why can’t I wear mine? I pushed the questions, okay, but why? I’m asking the questions, and I don’t mind pushing just a little bit.*

Tarana, a Kindergarten through 3rd grade Black special education teacher, also mentioned that expressing her identity through her hair requires patience because she needs to handle people’s invasion toward her hair.

*I do change my hair a lot. And I’ll do different hairstyles and very traditional braids… that comes with a lot of questions, but also a lot of invasiveness where people feel like it’s okay to just walk up and start petting my head… During those times, I can’t get upset… I have to be very patient and kind and just explain to them like that’s not appropriate behavior.*

Taylor, a 9th through 12th grade non-binary and transgender gay Spanish teacher, also commented how the ways Taylor dresses and puts on makeup shows the “desire to be more myself than I would be otherwise.”

*I’m regularly in a dress. If you saw me, you would see a man in a dress in heels most days. I, occasionally do makeup… But I am open as a drag queen at school. I do, drag events that I invite students and parents too, and I’m actually doing one this weekend that is really fun.*

This embodied expression of identities was more complex when they have multiple intersecting marginalized identities, such as George, a Latino gay instructional coach.

*I went through a stage where… I had earrings. I wore eyeliner. I used to wear, girls’ clothes, and I don’t mean girls as in dresses, I mean, like, sweaters that were nice sweaters, but guys could wear them. I would shop at Express. Just things like that. And then my Mexican side, I’d wear boots. I’d wear jeans. My hair was side and up, which was the style for Latinos in the 90s. So there were very specific things that you did to express your sexuality and your ethnicity. I still have some of those. No, I don’t have any more earrings, no, I don’t do any more eyeliner, but I still wear plaid. That was a big thing in the Latino culture, a lot of plaid. I still wear my jeans, I still wear boots. So my Latino culture is still very ingrained in daily living.*

Embodying their identities and expressing who they are in their space may demand extra emotional effort. However, these marginalized teachers strive to make their hidden identities more visible within school environments by intentionally and confidently expressing themselves and acting as their true selves.

#### 4.1.4. Contexts and Resources for Resistance: Social, Educational, and Historical Support

All marginalized teachers noted that they drew their strength to resist from multiple resources and contexts. At the macro level, societal and political movements provided them with the courage and affirmation they needed. Following the George Floyd protests, Amira recognized the importance of collective resistance and began organizing for change.

*After what happened with George Floyd, and after all the protests and everything, they just got to talking… They were like, we need to do something. So they basically emailed all the teachers of color… there was about, I want to say, like six or seven of us who were like, yes, we are interested.*

While Amira started organizing teacher groups instigated by the George Floyd protest, Edenia noted how the murder of George Flyod influenced her to address Black Lives Matter in her classroom.

*One of the very first things that we talked about at the beginning of the year was I wanted to make sure my students understood that Black Lives Matter is not a political statement. It is literally asserting that Black Lives Matter, and I needed my Black students to hear that, but I also needed my White students to hear that as well. So we did a lot of unpacking because it was a really tough summer.*

In relation to the Black Live Matter movement, Alma and Amira specifically addressed what the master narratives were, and why they resisted it.

*It [Black Lives Matter] makes me feel like I have a huge responsibility. … I work with teachers that don’t want to talk about things like that, or they think that, oh, the kids aren’t ready for this. They’re not developmentally there. They’re, just because, like, they’re nine, but, we could still have conversations we could they, they’re aware of some things, you know, they’re not oblivious, especially like, because they’re, they’re Brown, like, they know what’s going on.*

(Alma)

*Okay, like, this is happening in the world. I’m not going to shy away from talking about these things. Like, it is even more important to talk about these things… I was like, you know, it’s important for you to understand the power players in order to be a functioning member of society, because if not, you’re just gonna like be a little sheeple.*

(Amira)

At a more local and school level, colleagues’ support also encouraged them to enact pedagogical resistance by implementing an inclusive curriculum about women in STEAM. Tarana noted,

*Those teachers that went above and beyond to make me feel welcomed and make me feel seen. We’ve all been heard, I really connected with them and so when I’m in the classroom myself as a teacher, I really strive to be that safe place for other students.*

Gustavo, a 6th through 8th grade, Latino gay English learner teacher who incorporated books that address LGBTQ+ communities in teaching, also noted how comments from students’ parents affirmed his approaches to teaching, which made him realize, “oh, it’s okay, it’s okay. What I’m doing, you know, it’s okay”.

*One time another parent came up to me. And I was very scared about what she was going to say because it was June. And that was reading my literature. And she comes up to me with that face, that very stern face and I was worried for the worst, and she goes, can I tell you something and I go, what? I just want to say thank you. And I go why? Well, my brother has HIV and I don’t know how I would have brought it up with my daughter if it wasn’t for your books. So I opened the door for her to talk to her daughter. So that was another great moment.*

These supports and affirmation marginalized teachers addressed were not limited to their current transactions in schools and societies. They noted that their historical backgrounds and cultural heritage were also their sources of strength. Amira was inspired by the legacy of her ancestors, which allowed her to challenge present-day inequities.

*I have learned to live my life in honor of him and honor of my ancestors and honor all the people who couldn’t do the things that I get to… So I try to tell my students, like, you know, hold on, because you do have the power of your ancestors behind you.*

Amira also communicated this idea with students by creating pencils that displayed a quote, “struggle for your ancestors”, which provided her opportunities to discuss historical oppression and cultural values with students. Similarly, Raven, a Black special education teacher, emphasized her ancestral roots as her source of strength.

*My ancestors didn’t have an option to not go in the corn fields enough to pick it or to go in the fields to go in the rice fields, like they had to do that no matter if they just had had a baby or not. I’ve seen my mom moved my mom, I lost a brother and my mom literally went to work the next day. She could have stayed home, but that’s what she had to do. And she had to take care of five kids. So it’s the same thing with me. I have to take care of five kids, they live and breathe because I’m taking care of them. Think it has a lot to do with my race and where I come from.*

This sentiment was well-summarized in Tarana’s comments.

*I can’t turn off being black, I can’t change who I am, I have to really learn to thrive and excel, regardless of what’s going on. And so it’s definitely a part of me, and it’s something that I’m proud of, because of that reason, it teaches me to be resilient.*

## 5. Discussion

In this study, we argue for reconceptualizing resilience as a collective, socially embedded, and politically engaged process of resistance (Hernández-Johnson et al., 2023; Robinson & Schmitz, 2021), rather than as an individual trait of perseverance or strength (Tait, 2008). Dominant narratives often address marginalized teachers for merely “surviving” within oppressive systems but fail to interrogate the structures that necessitate such survival in the first place (Duggan, 2003; Halberstam, 2011). A critical approach instead centers resilience as relational and transformative, rooted in histories of struggle, communal knowledge, cultural wealth, and the pursuit of justice.

We observed that participants enacted resistance not simply as a reactive stance, but as an embedded, daily experience through which they navigate and challenge systemic oppression in their professional lives. In addition to this political nature, these acts of resistance also support core dimensions of teacher wellbeing. By asserting agency in decision-making for curriculum choice and instructional practices, teachers fulfill their psychological need for autonomy (Deci & Ryan, 2008). Also, speaking out against marginalization and oppression diminishes teachers’ emotional fatigue associated with silence and invisibility, thereby maintaining their sense of competence while alleviating their burnout as educators (Skaalvik & Skaalvik, 2010). Meanwhile, resistance also cultivates belonging and relational connection through alliances with students, colleagues, and communities, which is essential to teachers’ wellbeing and retention (Wator et al., 2025). It was through resisting inequity and asserting identity that teachers were able to experience emotional nourishment rather than depletion, thus employing resilience as a wellbeing-sustaining practice.

Findings also revealed the sources of collective and cultural strength (e.g., community, critical consciousness, ancestral knowledge, and radical pedagogy) that fueled resistance and reshaped resilience as a political and justice-oriented practice. These strengths did not only function as coping resources but also acted as emotional anchors that affirmed teachers’ identities and reminded them of broader historical contexts of resistance. Moreover, community connections offered mutual care, which eased teachers‘ experiences of isolation, and critical consciousness equipped teachers with language to name inequities rather than internalizing blame, all of which protected their psychological wellbeing. Ancestral and cultural knowledge also provided a sense of purpose and continuity, functioning as a protective factor against the risks marginalized teachers might face. The sources of collective and cultural strength, therefore, contributed to sustaining teachers’ wellbeing as well. In short, we reframe resilience as a capacity that entails resistance, which can be regarded as both a survival and wellbeing-enhancing practice. By doing this, this study contributes to expanding understandings of resilience beyond coping with and adapting to current contextual inequities and emphasizes the proactive and transformational deeds of confronting the oppressions.

This shift explicitly demonstrates that resilience must be reconceptualized to include teachers’ need and capacity to resist. Our findings show that resilience for marginalized teachers is not a passive process of endurance but an active form of opposition that simultaneously sustains wellbeing and advances equity. Rather than viewing resilience as an individualized capacity to simply “bounce back” from adversity (Price et al., 2012), participants in our study enacted resilience through intentional acts of resistance against systemic inequities. They exercised agency in multiple ways, including designing curricula that foster critical consciousness, adopting inclusive teaching practices, confronting prejudice from colleagues or school leaders, and confidently expressing their identities. Teachers intentionally designed or adapted curricula to challenge societal norms and raise students’ awareness of inequities, demonstrating that resistance can be embedded in everyday teaching practices. They also strengthened competence by prioritizing student wellbeing and belonging, even when advocacy led to tension or criticism from colleagues. The physical expression of identity, such as maintaining natural hair and wearing outfits that are aligned with their gender identity, further reflected their intentional and authentic resistance, which also reduced stress, supported psychological safety, and cultivated their relatedness through building cultural and identity-related connections with students, colleagues, and communities. These acts illustrated that resilience is not simply survival but the creation of space for equity, belonging, and justice within oppressive contexts, as well as the wellbeing-enhancing practice that enables teachers to sustain autonomy, competence, and relatedness in inequitable contexts (Deci & Ryan, 2008). This aligns with Robinson and Schmitz’s (2021) argument that resilience for marginalized communities must be understood as resistance, as it involves intentional opposition to injustice.

Another critical insight is the collective nature of resistance. In our study, teachers’ resistance was supported by broader social movements, colleague support, and cultural or ancestral strength. Societal and political movements provided affirmation and courage, while cultural heritage and historical experiences reinforced resilience. These findings suggest that resilience is not only an individual capacity but also a socially supported, community-driven form of resistance. This aligns with some previous studies indicating that resilience is socially embedded and historically situated, and from a critical consciousness framework, resistance highlights both individual and collective acts to resist oppressive systems, stigma, and violence (Ward, 2007). These collective practices also fulfilled teachers’ needs for relatedness and competence, which are the essential dimensions of wellbeing (Deci & Ryan, 2008). Meanwhile, as findings show that participants’ resistance practices were deeply tied to their cultural, racial, gender, and sexual identities, such as embracing natural hair, wearing identity-showing clothes, and integrating ancestors’ legacies into classroom discourse, resistance can be regarded as an identity-affirming practice that sustains teachers’ sense of self in the profession as well. Intertwined with identity affirmation and formation, resistance allows teachers to reduce alienation and enhance their emotional and professional wellbeing.

### 5.1. Limitations and Future Research

While this study provides valuable insights into how marginalized teachers enact resistance in resilience, it is important to acknowledge its limitations.

First, the sample size is relatively small and does not reflect the full range of marginalized teachers’ diverse backgrounds, which may limit the transferability of findings across broader contexts. In particular, participants of this study (e.g., Teachers of Color, LGBTQ+ teachers) represent a subset of marginalized identities. Other groups, such as immigrant teachers and teachers with disabilities, may engage in resistance in different ways. Although in-depth interviews allowed for rich, contextualized understandings of teachers’ lived experiences, future studies with larger and more diverse samples are needed to further refine and extend this reconceptualization of resilience.

Second, this study employed one type of data at one point in time: semi-structured interviews. Incorporating multiple data sources (e.g., classroom observations, student feedback, and interviews with school administrators) through repeated data collection points would provide methodological triangulation and enhance trustworthiness. Thus, future research can expand data sources and follow up with the participants over time, which would enable researchers to capture the multifaceted aspects and changing trajectories of resistance, exploring how teachers construct, adjust, and maintain their resistance strategies over time.

### 5.2. Practical Implications

By reconceptualizing resilience as resistance, this study highlights an urgent need for teacher education programs, school administrators, and policymakers to recognize resistance as an important path to wellbeing, particularly for those navigating systemic oppression. Supporting marginalized teachers involves shifting from simply resilience as coping to implementing strategies and interventions that affirm identity, cultivate agency, sustain wellbeing, and promote justice. (Deci & Ryan, 2008).

Teacher education and professional development programs should explicitly incorporate identity development, critical consciousness, and practical training as part of resistance-building, which helps both pre-service and in-service teachers identify and analyze systemic inequities in school (Cochran-Smith, 2004; Francis & Le Roux, 2011). Additionally, through professional learning, communities can serve as safe spaces for marginalized teachers to gain strength for resistance, where they share experiences, discuss resistance strategies, and collaborate on advocacy efforts. Such programs and spaces allow marginalized teachers to sustain autonomy, belongingness, and competence, which are essential components of wellbeing (Deci & Ryan, 2008).

Moreover, school administrators should establish structural support that protects marginalized teachers from isolation and retaliation. These may include mentorship networks, identity-affirming groups, conflict resolution systems, and equitable policies. Leadership that advocates for teacher agency and belongingness while disrupting discriminatory practices plays a critical role in preventing burnout and thus sustaining wellbeing of marginalized teachers (Collie & Martin, 2017; Skaalvik & Skaalvik, 2011).

At the system level, policies should not only emphasize diversity hiring but also commit to retention, leadership access, and wellbeing protections for marginalized teachers. By providing resources to build supportive school and community environments, developing anti-oppressive curricula, and taking other actions against educational inequalities, resistance can further serve as a catalyst for educational reform. Recognizing resistance within policy discourse, this perspective regards teachers as agents of equity whose wellbeing is intrinsically linked to the overcoming of systemic barriers. This approach thus contributes to fostering the advancement of the educational environment towards greater equity, inclusivity, and empowerment.

## 6. Conclusions

In conclusion, this study demonstrates that resilience for marginalized teachers must be reframed to include their capacity to resist. Such resistance is a proactive, collective, socially embedded, and politically engaged practice that directly contributes to sustaining wellbeing. Rather than merely enduring adversity, participants enacted resilience through intentional acts of resistance that affirmed identity, preserved agency, and cultivated belonging. These findings highlight that wellbeing is not achieved by resilience alone, but through resistance, where confronting inequities becomes a pathway to emotional and professional wellbeing. This reconceptualization of resilience has important implications for teacher education and policy. Moving beyond a narrow focus on cultivating individual coping traits, institutions must invest in structural supports, including justice-oriented preparation programs, mentoring networks, and inclusive curricula, to empower teachers to resist contextual oppressions and affirm their contributions. By expanding the conceptualization of resilience to include teachers’ capacity to resist, we can foster educational spaces that not only promote equity but also sustain the wellbeing of both teachers and students.

## Figures and Tables

**Table 1:** Participants’ Background Information.

Pseudonym	Race	Gender	Sexuality	Subject Matter
Alma	Hispanic	Female	Bisexual	All subjects (4th)
Amira	Afro-indigenous Latina	Cisgender Female	Heterosexual	Math (9th–12th)
Edenia	Latina	Cisgender	Lesbian	All Subjects (4th)
George	Latino	Cisgender Man	Gay	Instructional Coach
Gustavo	Latino	Man	Gay	English Learner, EL (6th–8th)
Jelani	Black	Woman	Bisexual	Social Science (9th)
Makala	Black	Woman	Heterosexual	English (11th &12th)
Magali	Dominican American	Woman	Bisexual	English Language Arts (4th)
Reina	Black	Woman	Heterosexual	Math (3rd–5th)
Raven	Black	Woman	Heterosexual	Special Education
Riley	Black	Woman	Heterosexual	Special Education (3rd–5th)
Shanika	Black	Woman	Heterosexual	ELA (6th–8th)
Sanaa	African American	Woman	Heterosexual	ELA (3rd–5th)
Tamia	African American	Woman	Heterosexual	Math, Science, Social Studies (Kindergarten—5th)
Tarana	Black	Woman	Heterosexual	Special Education (Kindergarten—3rd)
Tayla	Black	Woman	Heterosexual	Pre-K
Taylor	White	Man	Transgender and non-binary	Spanish (9th–12th)

Note. Race, gender, and sexuality labels are participants’ self-description.

## Data Availability

The datasets presented in this article are not readily available because the data are part of an ongoing study. Requests to access the datasets should be directed to Ji Hong.
